# A systematic review on risk factors and reasons for e-cigarette use in adolescents

**DOI:** 10.18332/tid/196679

**Published:** 2025-01-15

**Authors:** Víctor José Villanueva-Blasco, Lorena Belda-Ferri, Andrea Vázquez-Martínez

**Affiliations:** 1 Faculty of Health Sciences, Valencian International University, Valencia, Spain; 2 Valencian International University, Valencia, Spain

**Keywords:** adolescents, electronic cigarettes, risk factors, reasons for use, smoking cessation

## Abstract

**INTRODUCTION:**

The aim was to establish EC use risk and protective factors, the reasons for use, associations with tobacco and other substance use, and use for smoking cessation.

**METHODS:**

A systematic review following PRISMA guidelines was registered in PROSPERO (CRD42024532771). Searches in Web of Science and PubMed/MEDLINE (March–April 2024) used terms like ‘electronic cigarette’ and ‘adolescents’ with a PICO framework. Inclusion criteria covered studies on adolescents aged 10–19 years, published in English or Spanish (2018–2024). Three reviewers independently screened studies, achieving 96% inter-rater reliability. Data extraction followed standardized tables, and methodological quality was assessed using MMAT and GRADE tools, ensuring a robust evaluation of evidence on adolescent electronic cigarette use.

**RESULTS:**

From 895 studies, 50 met the inclusion criteria. The strongest risk factors for adolescent EC use include social acceptance and use within peer or family circles (13 studies), male gender (10 studies), low risk perception (6 studies), younger age (3 studies), and greater financial resources (3 studies). Motives for use include low perceived risk and appealing flavors, supported by both longitudinal and cross-sectional studies. EC use is significantly associated with smoking initiation (7 studies), cannabis consumption (8 studies), and alcohol use (4 studies). Evidence on the effectiveness of ECs as harm reduction or smoking cessation tools in adolescents remains inconclusive, with some cross-sectional studies supporting their utility and others finding contrary evidence. High-quality research indicates ECs are predominantly used alongside traditional cigarettes, with dual nicotine consumption patterns commonly observed.

**CONCLUSIONS:**

Evidence on adolescent EC use identifies risk factors, motives, and links to substance use. However, its role in harm reduction and smoking cessation remains insufficient and controversial. High-quality research is needed, as most studies are low quality. Targeted prevention strategies addressing social influences, perceptions, and accessibility are crucial to reduce adolescent EC use.

## INTRODUCTION

Previous studies claim that risk factors for adolescent e-cigarette use are similar to those for conventional tobacco^1^. In fact, most adolescents who use ECs also smoke conventional tobacco^2^. Previous research establishes smoking and other substance use^3^, parental use^4^ and peer group smoking^5,6^ as EC use risk factors.

EC users refer to a series of benefits that, from their perspective, justifies its use. Compared to cigarettes, they highlight that it tastes better, they get more effect with less quantity, and it is ultimately cheaper^7^ , more discreet, allowing for less socially stigmatized use^8,9^; it bothers those around them less^7^; it may be more accepted in the urban sensory landscape particularly in smoke-free spaces^10^; and it presents a ‘cool’ image^11^. Among adolescents, the main reasons for experimenting with ECs include curiosity, appealing flavors, consumer friends, and perceived benefits compared to cigarettes^12-14^, due to the belief that ECs are less harmful^14^ and help stop smoking^15^. This leads to a decrease in perceived risk of these devices relative to conventional tobacco.

The perception of risk attributable to adolescent drug use is related to the actual likelihood of drug use, and this is also true for ECs^16^. Numerous studies confirm that adolescent EC use is associated with later smoking^17-24^. Thus, ECs have been suggested as a gateway to smoking^18-27^.

There is also evidence of associations between EC use and alcohol consumption^3,28^; marijuana use^28-31^; consumption of other illicit drugs^3,29-31^; and use of prescription and non- prescription medications^3,30,31^.

Some studies show that ECs may be a potentially less toxic alternative to traditional cigarettes^32,33^. From a harm reduction perspective, EC use could help reduce or quit smoking^34,35^. However, evidence on the clinical utility of ECs for smoking cessation is limited and should be taken with caution, as this effect has been observed in a very small number of controlled clinical trials^36^. Unlike adult users who may use ECs, adolescent users are more likely to switch from ECs to conventional cigarettes^37^.

Despite all the accumulated evidence against EC use in the Spanish adolescent population, epidemiological studies report a prevalence of 44.3% for some lifetime use and 22.8% for the last 12 months^38^. These figures are far from the 5% smoking prevalence targeted by endgame strategies, despite the fact that the percentage of users has decreased as a result of the strategies implemented by the WHO (Framework Convention on Tobacco Control in 2003 and the MPOWER strategy in 2008)^39,40^. It is evident that these measures are insufficient to prevent the use of tobacco and related products such as ECs, probably due to the strategies used by the industry^41^, many of which are oriented to the adolescent and youth population. It may also be due to the lack of implementation of some of these measures in Spain^42,43^.

Thus, the research question is: ‘What are the risk and protective factors for e-cigarette use among adolescents, its association with tobacco and other substance use, and the evidence of its use as a smoking cessation strategy?’. The aim of the study was to establish the existing evidence in the adolescent population on: 1) EC use risk and protective factors; 2) motives for use; 3) the association of EC use with the consolidation of tobacco and other substance use; and 4) the evidence on EC use as a smoking cessation strategy.

## METHODS

### Search strategy

A systematic review was conducted following the guidelines of the PRISMA Statement (Preferred Reporting Items for Systematic reviews and Meta-Analyses)^44^ (Supplementary file). The search strategy was applied from March to April 2024. The database used was the Clarivate Analytics Web of Science (WoS) core collection and PudMed/MEDLINE. It was registered in the PROSPERO database (CRD42024532771). Two investigators (V-B and B-F) independently and blindly identified and screened titles and abstracts obtained from the electronic searches.

The search string included the terms electronic cigarette, e cigarette and adolescents, teenagers using the Boolean operators AND and OR resulting in the following canonical search equation: (‘electronic cigarette’ OR ‘e-cigarette’) AND (‘adolescents’ OR ‘teenagers’). To conduct the literature search, we followed the PICO framework (Population, Intervention, Comparison, and Outcomes) with: P=Adolescents; I=Electronic cigarette; C=Conventional cigarette; O=Risk factors, protective factors, motives for use, comorbid consumption, harm reduction, smoking cessation.

### Inclusion criteria

The inclusion criteria were: 1) studies that assessed EC use in adolescents aged 10–19 years; 2) in English or Spanish; 3) published in WoS or PudMed/MEDLINE; 4) access full text; 5) quasi-experimental studies and randomized controlled trials; 6) cross-sectional and longitudinal studies; and 7) published from 2018 to April 2024. The decision to include studies from 2018, took into account the considerable evolution of ECs over the past 5 years, examining different characteristics that may lead to distinct motives for use and consumer experiences; also, their potential effectiveness or ineffectiveness as harm reduction strategies and for smoking cessation. For these reasons, it was decided to consider studies from 2018 to 2024.

**Figure 1:**
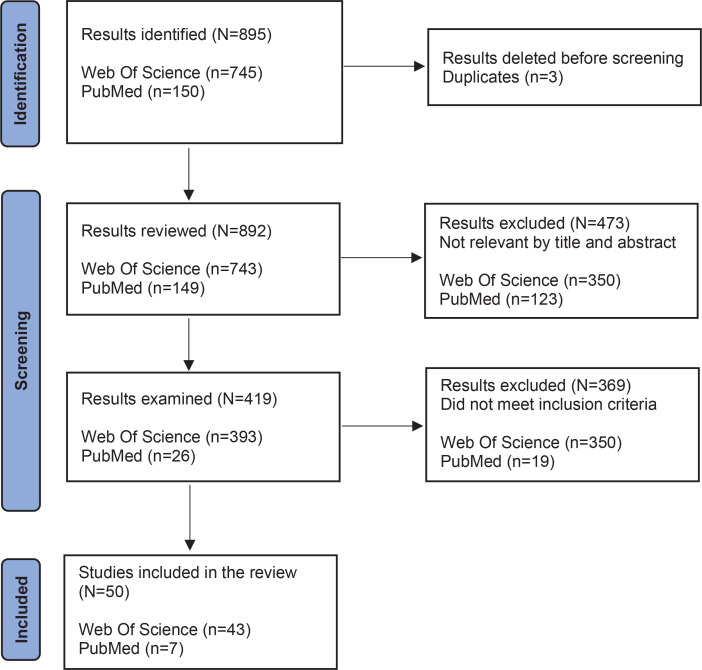
Diagram of the process followed based on the PRISMA model

As for exclusion criteria (Figure 1), we removed: 1) articles published in journals not indexed in WoS or PudMed/MEDLINE; 2) systematic reviews, meta-analyses, case or narrative studies, opinion articles, editorials, books, book chapters, practical guides, editorials and communications at congresses; 3) studies focused on variables other than those under study; 4) incomplete texts or written in a language other than English or Spanish; 5) studies on tobacco consumption by heat vaporization (e.g. Juul); and 6) articles published before 2018.

### Selection process

Three authors independently evaluated titles and abstracts to assess whether they met the inclusion criteria. The inter-rater reliability was 96%. Discrepancies were resolved by critical discussion until 100% agreement was reached. Each author then individually assessed the full text of the articles.

### Data extraction

Two authors (V-B and B-F) independently extracted data from each study in custom tables, including the following data: 1) authors; 2) type of study; 3) sample used; 4) objectives; and 5) main results and conclusions.

### Assessment of methodological quality

We assessed the methodological quality of the selected articles using the mixed-methods appraisal tool (MMAT) scale^45^. The MMAT is a critical appraisal tool designed for systematic reviews that has five items that assess sampling strategy, representativeness of the sample, adequacy of measurements, risk of non-response bias, and adequacy of statistical analyses. Additionally, a GRADE evidence assessment was conducted following the recommendations of Aguayo-Albasini et al.^46^.

## RESULTS

### Study selection

The study selection process is shown in Figure 1. The first search yielded 895 results related to EC consumption in adolescents. After reviewing the title and abstract, 473 results were excluded. In the second step, after reviewing the topic content and appropriateness and the application of the inclusion and exclusion criteria, 369 results were removed. Finally, 50 studies remained and form the basis of the present systematic review. Figure 1 shows the selection procedure.

To assess the methodological quality of the selected articles, the MMAT scale^45^ was applied (Table 1). All studies met 60% of the criteria, with 85.60% being the mean percentage of criteria met. Table 2 shows the information on the 50 studies including author, type of study, sample, objectives, main results and conclusions. A summary of the main findings is presented below.

**Table 1:** Methodological quality assessment of the studies included in the systematic review

*Authors*	*P1*	*P2*	*P3*	*P4*	*P5*	*Percent compliance*
Bowe et al.^47^	Yes	Yes	Yes	Yes	Yes	100
Chang and Seo^48^	Yes	Yes	Yes	Yes	Yes	100
Conner et al.^49^	Yes	Yes	Yes	Yes	Yes	100
Dai et al.^50^	Yes	Yes	Yes	Yes	Yes	100
Fan et al.^51^	Yes	Yes	Yes	Yes	Yes	100
Kinnunen et al.^52^	Yes	Yes	Yes	Yes	Yes	100
Tarasenko et al.^53^	Yes	Yes	Yes	Yes	Yes	100
Van Minh et al.^54^	Yes	Yes	Yes	Yes	No	80
Wamba et al.^55^	Yes	Yes	Yes	Yes	Yes	100
Zavala-Arciniega et al.^56^	Yes	No	Yes	No	Yes	60
Durkin et al.^57^	Yes	No	Yes	No	Yes	60
Usidame et al.^58^	Yes	Yes	Yes	Yes	Yes	100
Conner et al.^59^	Yes	Yes	Yes	Yes	Yes	100
Zhao et al.^60^	Yes	No	Yes	Yes	Yes	80
Carey et al.^61^	Yes	Yes	Yes	No	Yes	80
El-Amin et al.^62^	Yes	Yes	Yes	No	Yes	80
Monzón et al.^63^	Yes	Yes	Yes	Yes	Yes	100
Tudor et al.^64^	Yes	No	Yes	Yes	Yes	80
Ahuja et al.^65^	No	No	Yes	Yes	Yes	60
Hunter et al.^66^	Yes	Yes	Yes	Yes	Yes	100
Liu et al.^67^	Yes	No	Yes	Yes	Yes	80
Vogel et al.^68^	Yes	No	Yes	No	Yes	60
Vrinten et al.^69^	Yes	Yes	Yes	Yes	Yes	100
Ollila et al.^70^	Yes	Yes	Yes	Yes	No	80
McCabe et al.^71^	Yes	Yes	Yes	Yes	Yes	100
Kinnunen et al.^72^	Yes	Yes	Yes	No	Yes	80
Staff et al.^73^	Yes	Yes	Yes	Yes	Yes	100
Vogel et al.^74^	Yes	No	Yes	No	Yes	60
Thoonen and Jongelis^75^	Yes	Yes	Yes	Yes	No	80
Leventhal et al.^76^	Yes	Yes	Yes	Yes	No	80
Davis et al.^77^	Yes	Yes	Yes	Yes	Yes	100
Thoonen et al.^78^	Yes	Yes	Yes	Yes	Yes	100
Jongelis et al.^79^	Yes	Yes	Yes	Yes	Yes	100
Lechner et al.^80^	Yes	No	Yes	No	Yes	60
Chaffe et al.^81^	Yes	No	Yes	Yes	Yes	80
Evans-Polce et al.^82^	Yes	Yes	Yes	No	Yes	80
Kinnunen et al.^83^	Yes	No	Yes	No	Yes	60
Yang et al.^84^	Yes	No	Yes	Yes	No	80
Azagba^85^	Yes	Yes	Yes	Yes	Yes	100
Owotomo et al.^86^	Yes	Yes	Yes	No	Yes	80
Audrain-McGovern et al.^87^	Yes	Yes	Yes	No	Yes	80
Azagba et al.^88^	Yes	Yes	Yes	Yes	Yes	100
Bentivegna et al.^89^	Yes	Yes	Yes	Yes	Yes	100
Duan et al.^90^	Yes	Yes	Yes	Yes	Yes	100
Evans-Polce et al.^91^	Yes	No	Yes	No	Yes	60
Park et al.^92^	Yes	No	Yes	No	Yes	60
Wang et al.^93^	Yes	Yes	Yes	Yes	Yes	100
Case et al.^94^	No	Yes	Yes	Yes	Yes	80
Foxon et al.^95^	Yes	Yes	Yes	Yes	Yes	100
Trucco et al.^96^	Yes	No	Yes	No	Yes	60

P1: Is the sampling strategy relevant to address the research question? P2: Is the sample representative of the target population? P3: Are the measurements adequate? P4: Is the risk of non-response bias low? P5: Is the statistical analysis adequate to answer the research question?

**Table 2:** Description of the studies selected about EC use risk and protective factors, and motives for use

*Authors*	*Study type*	*Sample*	*Objectives*	*Main results and conclusions*
Bowe et al.^47^	Cross-sectional study	N=4150 Irish adolescents aged 15–16 years	To describe the epidemiology of current just EC use, just conventional cigarette smoking, and dual use and to examine and compare family, peer group, and individual factors associated with these behaviors.	The risk profiles of exclusive EC users differ from dual users and conventional cigarette users. Compared to women, men were twice as likely to use EC or dual use. Having a parent who smoked increased the likelihood of using any nicotine product. Dual use is the most common behavior among adolescent users of nicotine products. Average and below average performers were more likely to use nicotine products. Strong statistically significant association between peer-related factors and use of all nicotine products. ECs may appeal to a population that would engage in positive lifestyle behaviors who would not use conventional cigarettes.
Carey et al.^61^	Longitudinal cohort study	N=3907 American students aged 11–16 years	To identify risk factors (risk perceptions, social influences and norms, affective and behavioral risk factors) associated with susceptibility to EC use during a one-year follow-up among adolescents with no previous experience with EC.	Different risk factor profiles: - 6th graders (11-12 years): EC use among family members influences the transition to becoming susceptible. - Students in 6th grade (11–12 years) and 8th grade (13–14 years): Positive affect as a protective factor - 8th graders (13–14 years): low school performance, beliefs that ECs are ‘okay to use’ and ‘commonly used‘ increase risk - 8th and 10th (15–16 years) graders: higher levels of sensation seeking increase risk.
Conner et al.^59^	Cluster randomized controlled trial	N=3210 English students (UK) aged 13–14 years at baseline and never used EC/cigarettes	To assess EC/cigarette predictors of usage patterns.	Friends who smoke, family who smoke, impulsivity and gender: predictors in all groups. Males: more likely to be in the EC-only group Higher levels of impulsivity: more likely to be in the smoking groups.
Conner et al.^49^	Randomized controlled trial	N=3289 Non-smoking English students aged 13–14 years Prospective study with 12 and 24 months’ follow-up	Check for differences in regular use/occasional use between different EC users groups: early users, late users, non-consumers.	Nearly half of smokers had one or more family members who smoked. Early users (EC use between the ages of 13 and 14) → higher rates of later initiation of cigarette smoking than late users (use between the ages of 14–15 years) → difference that can be attributed to a longer period of time to initiate cigarette smoking in early users.
Dai et al.^50^	Cross-sectional study	N=16123 Chinese adolescents aged 13–18 years	To examine the association between social environment exposure and EC use, as well as gender and school type differences.	Current EC use rates among boys were higher than those among girls. Adolescents’ current EC use was significantly associated with parental and peer EC consumption.
Davis et al.^77^	Longitudinal study	N=4875 American students aged 13–19 years	To examine the use of refreshing flavors and their association with EC use behaviors.	Those who vaped refreshing flavors reported higher frequency of vaping in the past 30 days and higher rates of electronic liquid nicotine use in the past 30 days than those who did not vape refreshing flavors.
Durkin et al.^57^	Cross-sectional study	N=562 American students aged 14–18 years	To investigate associations between costs and benefits perceptions, peer use, self-efficacy to resist use, and self-reported use.	Increased risk of current EC use → more friends who use ECs and when they have a more favorable reaction to ECs. Older adolescents: less likely to use EC currently. The likelihood of using CE is a function of peer use (social norms), expectations about costs and benefits (beliefs), and self-efficacy to resist (perceived control).
El-Amin et al.^62^	Longitudinal study	Finnish adolescents aged 12–18 years N=4058 in 2017 N=3520 in 2019	To explore if perceptions of harmful health effects of different tobacco products are related to consumption, school performance, parental smoking and socioeconomic status.	Adolescents perceive fewer negative health consequences from ECs than from conventional cigarettes. Those who have used tobacco do not perceive ECs as harmful to health as often as those who have never used them. Parental smoking was associated with their child’s view that cigarettes and other tobacco products are not harmful to health. Strong association between poor school performance and misperceptions about the harmfulness of cigarettes.
Fan et al.^51^	Cross-sectional study	N=12410 Secondary school students in China in 2019 N=12880 in 2021	To explore the factors influencing experimentation and use of EC by adolescents. To compare the vaping status of EC with different characteristics.	The main factors associated with EC use were: experimentation with cigarettes, male gender, higher weekly allowance, exposure to secondhand smoke at home, exposure to EC advertising, closest friends who smoke, belief that smoking makes young people appear more attractive, belief that tobacco helps young people feel more comfortable in social situations.
Hunter et al.^66^	Cross-sectional study	N=4204 Australian participants (mean age=15.70 years, SD=0.60)	To examine the associations between EC use, perceived peer EC use, and bullying.	Strong cross-sectional associations were found between EC use and perpetration of bullying, victimization from bullying, bullying victim status, and perceived peer use. Bullying perpetrators were three times more likely than non-perpetrators to have used an EC → Bullying perpetrators often experience difficulties in emotional regulation, externalizing behavior, and impulsivity, and are more likely to associate with delinquent peers and seek social dominance.
Jongelis et al.^79^	Cross-sectional study	N=4617 Australians aged 12–17 years	To investigate susceptibility to EC use among Australian adolescents who had never smoked cigarettes or used ECs.	45% of the sample who had never smoked or vaped were susceptible to future vaping. Important correlates of susceptibility included the belief that EC use is associated with positive affect regulation outcomes and helps with smoking cessation.
Kinnunen et al.^52^	Cross-sectional study	N=12167 European students aged 13–18 years (Netherlands, Portugal, Ireland, Germany, Italy, Belgium and Finland)	Identify the social correlates of EC use, and if they are the same for EC-only use, conventional cigarette-only use, and dual use.	EC use risk factors (especially dual use): male gender, older age, parental and peer smoking.
Lechner et al.^80^	Longitudinal study	N=1023 American students Average age=12.2 years. Survey every 6 months for two years and one at end of year	To examine the specific role of cognitive susceptibility to smoking prior to initiating use of any of the products, while controlling for other common risk factors.	Those who used any tobacco product perceived ECs as less harmful compared to abstainers. Beliefs that ECs have low addiction potential and are safer than cigarettes are associated with use.
Leventhal et al.^76^	Longitudinal study	American 11th and 12th grade students Wave 1, N=3251 Wave 2, N=3232 Wave 3, N=3078 Wave 4, N=3168 Wave 5, N=3140	To assess whether those using ECs with non-traditional flavors (fruits, sweets) were more likely to continue vaping and progress to more frequent vaping.	Adolescents who vaped ECs with non-traditional flavors, compared to those who vaped exclusively tobacco, mint, or menthol-flavored ECs, or unflavored ECs, were more likely to continue vaping and take more puffs per vaping occasion 6 months later → higher likelihood of continued vaping and progression to more frequent vaping patterns.
Liu et al.^67^	Cross-sectional study	N=1289 Taiwanese students aged 12–18 years	To identify potential factors related to EC use behaviors in adolescents.	Risk factors included tobacco consumption, use of other substances, EC use by close friends, attitudes of close friends toward EC use, and family environment.
McCabe et al.^71^	Cross-sectional study	N=38926 American students aged 13–18 years [8th Grade, N=10899 (13–14 years); 10th, N=10438 (15–16 years); 12th, N=17.589 (17–18 years)]	Identify EC use risk factors in relation to school characteristics (public vs private, urban vs rural, racial composition at the school level).	School environments serve as important risk factors for EC use at the individual level. Increased risk of EC use in females and males at higher grade levels. Males in schools with high levels of heavy alcohol and marijuana use are at higher risk for EC use compared to females in these same schools.
Monzón et al.^63^	Discrete choice experiment (DCE)	N=2028 Guatemalan students aged 13–18 years	To assess how tobacco product attributes (type of tobacco product, flavors, and nicotine content) influence their appeal among adolescents.	ECs appear to be more attractive and perceived as less harmful than heated tobacco products and cigarettes. Flavors enhance product appeal and influence perceptions of harmfulness.
Ollila et al.^70^	Cross-sectional study	N=98758 European students aged 15–16 years	To examine the associations between national regulations on ECs and their use, considering socioeconomic status, laws on tobacco sales age, and income level.	More comprehensive regulations on ECs were associated with a lower risk of exclusive EC use and dual use → perceived greater difficulty in obtaining them. Higher levels of parental education protected against regular and current smoking, EC use, and dual use. Perception of family background was associated with a higher likelihood of current exclusive and dual EC use.
Tarasenko et al.^53^	Cross-sectional study	European students from 17 countries aged 11–17 years	To estimate the crude and adjusted prevalence of EC use by sex and amount of disposable income.	There is a growing trend in EC use and initiation from the age of 11 years. Many more male students than female students reported EC use. Students with an average amount of disposable income were significantly more likely to use ECs than those with lower amounts.
Thoonen et al.^78^	Cross-sectional study	N=1497 Australian adolescents aged 12–17 years	To identify beliefs about the risks and benefits associated with the use of different types of ECs. To assess differences in beliefs according to age and user status.	71% believe that EC use helps smokers reduce their tobacco consumption. Adolescents were significantly more likely to hold favorable beliefs about the social and psychological benefits of EC use. Adolescents did not recognize the harms associated with nicotine-free and flavored ECs and maintained positive perceptions about EC use.
Thoonen and Jongelis^75^	Cross-sectional study	N=4617 Australians aged 12–17 years	To explore and compare reasons for intended use, initiation, and current use of ECs.	Adolescents in most user groups were more likely than adults to report using ECs out of curiosity and because they tasted good. Adults are significantly more likely than adolescents to report using ECs for smoking cessation or reduction purposes.
Trucco et al.^96^	Cross-sectional study	N=176 American adolescents aged 14–17 years and their parents	Examine the influences (positive expectations and intentions to use) of parents and peers in the early stages of EC use.	Parental influence can buffer the effect of peer norms and positive expectations on an adolescent’s decision to use EC.
Tudor et al.^64^	Cross-sectional study	N=748 Romanian students aged 13–14 years	To assess awareness, opinions, and practices regarding EC use, as well as factors associated with their use.	The main reason for trying ECs is curiosity. Peer influences correlated with EC experimentation. Beliefs that ECs help with smoking cessation and that they are less harmful strongly correlated with EC experimentation. No gender differences were found in EC use.
Usidame et al.^58^	Cross-sectional study	N=35456 American students aged 13–18 years [8th Grade, N=11189 (13–14 years); 10th, N=12882 (15–16 years); 12th, N=11385 (17–18 years)]	To assess sociodemographic predictors of exclusive EC use and dual use with smoking tobacco among adolescents.	The EC only usage pattern is most frequent across all grades and is most common among girls. Younger boys are less likely to exclusively use EC than younger girls.
Van Minh et al.^54^	Longitudinal study	N=3331 for 2013 N=7796 for 2019 Vietnamese adolescents aged 13–17 years	To compare the prevalence of smoking and associated factors.	Risk factors included male gender, mental health problems, self-reported experience of bullying, school absenteeism, alcohol consumption, and sedentary lifestyle. Protective factors included parental control and respect.
Vogel et al.^68^	Longitudinal study	N=173 American adolescents aged 13–18 years (consumed at least once in last 30 days and at least 10 times in their lifetime)	Identify correlates of EC use frequency and dependence.	Higher risk of problematic use: those with greater exposure to nicotine and greater acceptance of EC use in their social circle. EC use Risk profiles may differ from smoking cigarettes. Frequent use was associated with receiving the first e-cigarette from a family member, using nicotine in all ECs, and having more friends who use ECs. Dependence was associated with younger age at first use, recent cigarette use, and having more friends who use EC.
Vogel et al.^74^	Longitudinal study	N=173 American adolescents aged 13–18 years (consumed at least once in last 30 days and at least 10 times in their lifetime)	Examine the reasons to use and stop using ECs. Determine persistence in EC use and dual consumption and stability in frequency and dependence of EC use.	The three main reasons for initiating and continuing use: socialization, enjoyment and flavors. The top three reasons for quitting smoking: desire for self-improvement, difficulty maintaining the device, and getting in trouble for vaping at home or school.
Wamba et al.^55^	Cross-sectional study	N=7950 French adolescents aged 15–16 years	To assess the evolution of experimentation and use of tobacco and ECs.	Their motivations for experimenting with ECs are mainly associated with recreational leisure, but not with the desire to reduce or quit smoking. The prevalence of current smoking was higher in boys than in girls, as was the prevalence of current vaping.
Zavala-Arciniega et al.^56^	Cross-sectional study	N=951 Mexican students aged 12–13 years at baseline who have used in the past month	To assess the correlates of the frequency of EC use among Mexican students who currently use EC.	Being male, using drugs, and having family members who use ECs and cigarettes were associated with a higher frequency of EC use in the past month. Curiosity was the most common reason for using cigarettes.
Zhao et al.^60^	Case-control study	N=44 Chinese students aged 16–18 years who had used ECs and 44 who had not	To explore the characteristics and risk factors of EC use in adolescents.	Characteristics of EC use include: early age at first use, consumption of a large quantity, smoking in discreet places to hide from adults. Reasons for using ECs include: curiosity, desire to replace traditional cigarettes. Risk factors for EC use include: insufficient understanding of individual-level harm of ECs, peer influence at the interpersonal level, influence of social and environmental factors such as EC sales in stores, pleasant aromas, and multiple flavors.

**Figure 2:**
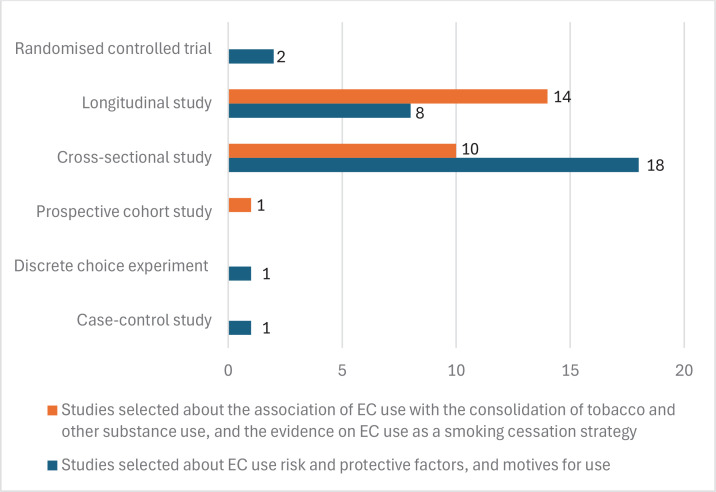
Design of the studies analyzed according to the topic addressed

In Figure 2, most studies are cross-sectional^47,50-53,55-58,64,66,67,70,71,75,78,79,96^, followed by longitudinal studies^54,61,62,68,74,76,77,80^. Among studies analyzing risk and protective factors, as well as motives for EC use, arranged from lower to higher quality in study design, there is one case-control study^60^, one discrete choice experiment^63^, 18 cross-sectional studies^47,50-53,55-58,64,66,67,70,71,75,78,79,96^, 8 longitudinal studies^54,61,62,68,74,76,77,80^, and 2 randomized controlled trials^49,59^. Among studies examining the association of ECs with tobacco use initiation and use of other substances, as well as evidence of ECs as a smoking cessation strategy in adolescents, arranged from lower to higher quality in study design, there is one prospective cohort study^61^, 10 cross-sectional studies, and 14 longitudinal studies. Only the 2 randomized controlled trials have a high level of evidence according to GRADE^46^ as they are randomized studies. The rest of the analyzed studies are observational, so their level of evidence is low or very low, according to GRADE^46^.

### Adolescent EC use risk and protective factors

Regarding individual risk factors, gender was identified as a predictor, with males being more likely to use ECs^47-56^. Concerning age, older adolescents (aged 17–18 years) were less likely to use ECs compared to younger adolescents (aged 14–15 years)^57^. However, the youngest adolescent males (aged 13–14 years) were less likely to use than the youngest adolescent females (aged 13–14 years) as these are more curious and susceptible to consuming ECs^58^. For their part, sensation seeking and higher levels of impulsivity are associated with a higher risk of EC use at a younger age^59^.

Regarding risk perception, adolescents consider the health risk to be low due to an insufficient understanding of the harm of ECs at an individual level^60^, and that the negative consequences are fewer than for conventional cigarettes^61-64^.

Among social factors, those adolescents with greater exposure to nicotine and greater acceptance and use of ECs in their social circles, such as family or friends, are at a greater risk for more problematic EC use^50-52,56,57,59,60,64-69^.

Other authors have found an increase in EC use among adolescents with higher disposable income^51,53,70^. A higher level of parental education, as well as greater parental control^54^, protects against smoking and EC use^70^.

Among school factors, school environments are closely related to cigarette and EC use. Boys from schools with high levels of alcohol and marijuana use were at higher risk of EC use compared to girls attending the same schools^71^. School absenteeism has also been shown as a risk factor in consumption^54^. On the other hand, excellent academic performance appears to be a protective factor for experimentation with ECs^72^.

Finally, Staff et al.^73^ have found positive links between EC use and maladjustment, and delinquency.

**Figure 3:**
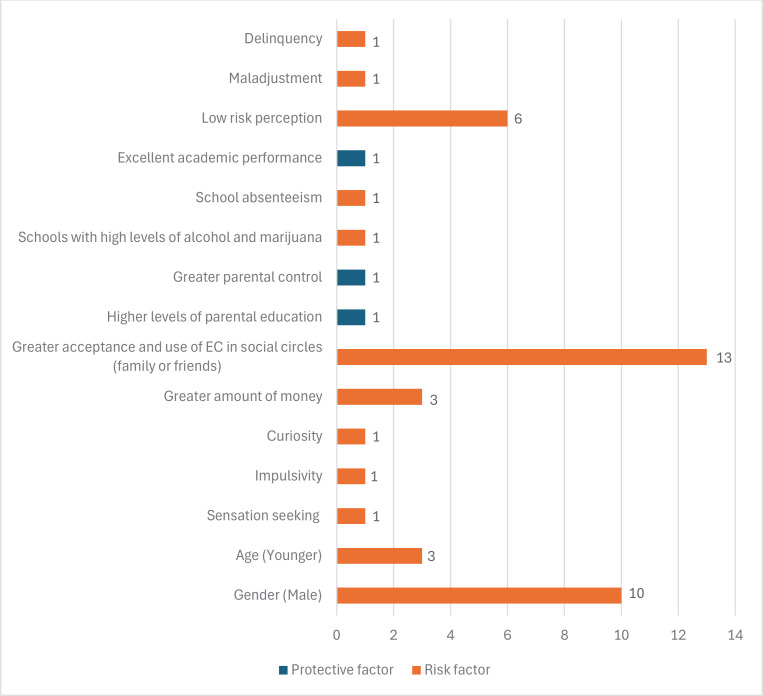
Number of studies analyzed that confirm each protection factor or risk related to the use of ECs

In Figure 3, the number of studies confirming each protective or risk factor related to EC use in adolescents can be seen. The risk factors with the strongest empirical support, presented based on the number of studies and the quality of the evidence according to GRADE^46^, are: 1) greater acceptance and use of ECs in their social circles (family or friends), reported in 13 studies, of which only one is of high quality^59^; 2) gender indicating that males show higher EC use, reported in 10 studies, of which only one is of high quality^49^; 3) low risk perception, reported in 6 studies, all of low quality; 4) age, with younger age associated with higher risk, reported in 3 studies, of which one is of high quality^49^; and 5) the availability of higher disposable income among adolescents, reported in 3 studies, all of low quality.

### Reasons for EC use in adolescents

Some of the main reasons for initiating and continuing EC use were socialization, enjoyment, flavors^74^, and curiosity^56,60,64,75^.

In terms of flavors, it has been found that adolescents who vaped non-traditional flavors (fruits, sweets, etc.) were more likely to continue vaping^76^. Refreshing flavors have been associated with higher rates of use and increased frequency of consumption^77^. Additionally, flavors have also been found to enhance the attractiveness of the product and influence perceptions of harm^63,75^.

Adolescents’ perceptions of negative health consequences are lower for ECs than for conventional cigarettes^61-64^, which favors their use. This positive perception of EC use includes favorable beliefs regarding the social and psychological benefits of their use^78^, as well as positive outcomes in affect regulation^79^.

In addition, those who used any tobacco product perceived ECs as less harmful compared to abstainers, with the belief that ECs have lower addiction potential and are safer than conventional cigarettes^80^. Adolescents also perceive that using ECs implies a low health risk^60^, which favors their use.

**Figure 4:**
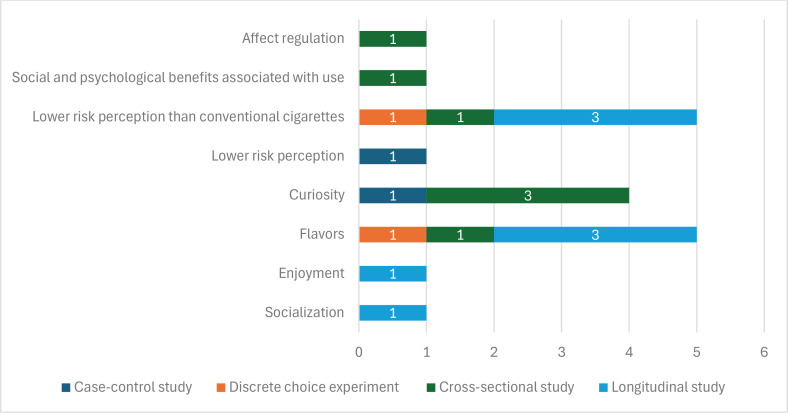
Number of studies analyzed that confirm each reason for EC use

In Figure 4, the number of studies confirming each motive for EC use in adolescents is presented, considering their experimental design. The motives for consumption with the strongest empirical support, based on the number and quality of studies, are low risk perception and flavors, both with three longitudinal studies and one cross-sectional study. All these studies are of low quality according to GRADE^46^.

### EC use, the consolidation of adolescent tobacco and other substance use

ECs may serve as a gateway to later conventional cigarette use^81-84^. Adolescents aged 13–14 years (early users) who use ECs have higher rates of subsequent cigarette smoking initiation than adolescents aged 14–15 years (late users) who report using ECs^49^. Frequent EC use was associated with a greater probability of cigarette smoking^85^. However, this increased risk was observed among adolescents who had no prior intention to smoke, but not among those who had previously expressed an intention to smoke^86^.

Vogel et al.^74^ indicate a significant increase in the percentage of daily users, frequency of use, nicotine exposure and dependence among adolescents who use ECs. It is also associated with future alcohol and marijuana use^48,87-93^, and non-prescription drug use^91^. Staff et al.^73^ have found positive links between EC use and other substance.

**Figure 5:**
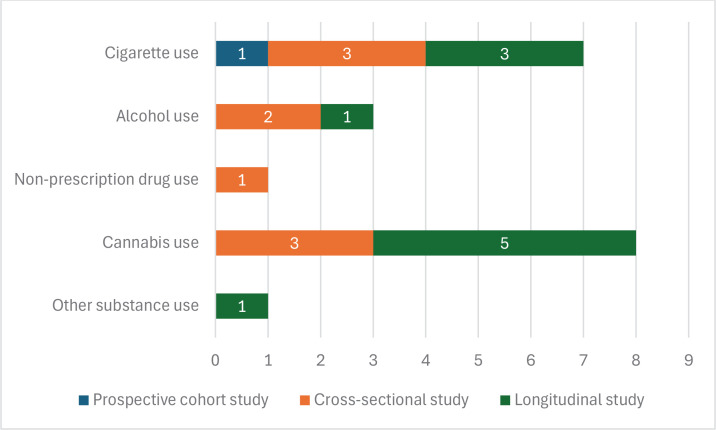
Number of studies analyzed that confirm the relationship between the use of EC and the consumption of conventional cigarettes and other substances

In Figure 5, the number of studies confirming the time^98^. As in previous literature regarding smoking, greater family acceptance and use of ECs as a risk factor for problematic EC use, or friendships with people who are more likely to use ECs^4,62,72^ or friendships^5,6^, is also confirmed in the investigations analyzed in the present study^50-52,56,57,59,60,64-69^. relationship between EC use and the consumption of conventional cigarettes and other substances is presented, considering their experimental design. The substances with the strongest empirical support, based on the number and quality of studies, are cannabis with five longitudinal studies^87,89,90,92,93^ and three cross-sectional studies^48,88,91^; followed by conventional cigarette consumption, with three longitudinal studies^72,83,84^, three cross-sectional studies^47,81,85^, and one prospective cohort study^86^. Alcohol consumption follows, with two cross-sectional studies^48,91^ and one longitudinal study^92^. All these studies are of low quality according to GRADE^46^, except Coneer et al.^49^, which provides high-quality evidence regarding the early use of ECs increasing the risk of conventional cigarette consumption.

### ECs as a smoking cessation strategy in adolescents

Dual use of ECs and conventional cigarettes is the most frequent behavior among adolescent users of nicotine products^45^. In the study by Vogel et al.^74^ none of the dual users abstained from both products at any of the follow-ups. Thus, for adolescents, ECs appear to be a complementary nicotine product to conventional cigarettes, and not a replacement^47,52^. Here, the use of ECs as a smoking cessation strategy is uncommon in adolescents^56,82,84^. Adolescents do not initiate EC use with the desire to reduce or quit smoking^55^, in fact ECs do not deter cigarette smoking habits and may foster it in this population^81^. Adults are significantly more likely than adolescents to report using ECs for smoking cessation or reduction purposes^75^. Adolescents who dual use may view ECs as less harmful and, therefore, may be less motivated to quit using them^94^. In addition, experiencing more EC-specific dependence symptoms was associated with lower odds of both wanting and attempting to quit in the past year^94^. However, Foxon et al.^95^ did find that ECs may have substantial implications for harm reduction among youth (Table 3).

**Table 3:** Description of the studies selected about the association of EC use with the consolidation of tobacco and other substance use, and the evidence on EC use as a smoking cessation strategy

*Authors*	*Study type*	*Sample*	*Objectives*	*Main results and conclusions*
Ahuja et al.^65^	Longitudinal study	N=243 exclusive EC users (last 30 days) among American adolescents	To examine the association of socioecological factors with smoking cessation behavior among adolescents.	Individual-level factors (perception of harm) and interpersonal factors (EC use at home and by significant others) may play a significant role in EC cessation behavior, decreasing the likelihood of quitting ECs.
Audrain-McGovern et al.^87^	Longitudinal study	N=2668 American students aged 14–15 years who never used marijuana (24-month follow-up)	To examine whether adolescent use of EC, hookah, or combustible cigarettes is associated with marijuana initiation and current use, as well as co-use of tobacco and marijuana.	Use of any tobacco products (EC, hookah, and combustible cigarettes) at the start of the study was associated with tobacco and marijuana use 24 months later → EC or hookah use in early adolescence more than doubled the odds of using both tobacco and marijuana in mid-adolescence. Evidence of a prospective relationship between adolescent EC use and narghiles and the risk of marijuana initiation and use.
Azagba^88^	Cross-sectional study	N=23429 Canadian students aged 14–18 years	To examine associations between EC use, dual use and frequency of cannabis use.	Sequential risk gradient: dual users at highest risk of cannabis use, followed by only cigarette users, only EC users with non-users as the lowest risk group. Clustering risk behaviors of EC users.
Azagba et al.^85^	Cross-sectional study	N=51662 Canadian students aged 12–18 years	To examine the frequency of EC use among cigarette smokers and non-cigarette smokers and the association between frequency of EC use and smoking.	Frequent EC use was associated with increased odds of cigarette smoking. EC use is more common among those who have occasionally smoked and in the past 30 days compared to adolescents who have never smoked.
Bentivegna et al.^89^	Longitudinal study	N=7551 American students aged 12–17 years N=328 occasional e-cigarette users, N=7223 never consumed	Examining the longitudinal relationship between EC and multiple substance use.	EC use associated with marijuana use, marijuana in electronic nicotine devices, over-the-counter medications (such as Ritalin/Adderall), and use of various substances, but not pain relievers, sedatives, or tranquilizers. ECs may encourage and facilitate additional risk-taking behaviors in young people. Initial EC use was associated with future use of multiple substances → more worrisome progression in youth who use ECs.
Bowe et al.^47^	Cross-sectional study	N=4150 Irish adolescents aged 15–16 years	To describe the epidemiology of current just EC use, just conventional cigarette smoking, and dual use and to examine and compare family, peer group, and individual factors associated with these behaviors.	Dual use may represent a transition to the use of tobacco products, a process known as the gateway effect.
Case et al.^94^	Cross-sectional study	N=2891 American students aged 12–17 years	Examine the elements related to dropout by use group (just EC users versus dual users). To examine the prevalence of nicotine dependence symptoms specific to ECs.	Dual teens may see EC as less harmful → less motivated to stop using. Experiencing more EC-specific dependence symptoms was associated with lower odds of both wanting to quit and attempting to quit in the past year.
Chaffe et al.^81^	Cross-sectional study	N=1295 American students aged 12–17 years who had smoked a cigarette (≥1 puff) but had not yet smoked 100 cigarettes	To evaluate the associations between EC use and progression to established smoking among adolescents who had already tried cigarettes.	Among adolescents who experimented with cigarettes, EC use was positively and independently associated with progression to established current smoking → ECs do not deter cigarette smoking and may promote it in this population. EC use is more likely to encourage youth to smoke than to deter them from smoking once they have already experimented with cigarette use.
Chang and Seo^48^	Cross-sectional study	N=14638 American students In age categories: 12–14 years 15–16 years 17–18 years	Examine whether EC use is related to other risk behaviors and whether age and gender play a role in these associations.	Moderating effect of age and sex on the association between EC use and certain risk behaviors → Older students and males more likely to use ECs. EC users are more likely to use other substances, including cigarettes, alcohol, and marijuana simultaneously.
Duan et al.^90^	Longitudinal study	N=19503 Americans aged 12–17 years who had never consumed cannabis (5049 in Round 1, 6566 in Round 2, and 7888 in Round 3)	To investigate whether the association between initial EC use and subsequent cannabis consumption differs depending on the state’s recreational cannabis legalization status.	EC use is associated with cannabis initiation among youth. In adolescents who had never consumed cannabis and lived in states that legalized recreational cannabis use for adults, EC use was significantly associated with a higher risk of subsequent cannabis initiation.
Evans-Polce et al.^91^	Cross-sectional study	N=717 American students aged 18–19 years (from 2014 to 2016 cohorts)	Examine associations of ECs with use of marijuana, alcohol, non-prescription drugs.	EC users: - Increased likelihood of using non-prescription drugs, other drugs and alcohol one year later. - Increased risk of cigarette and marijuana use Strong association between EC use and marijuana use.
Evans-Polce et al.^82^	Cross-sectional study	N=36506 American students aged 13–16 years	Examine patterns of use onset and compare with 2015, 2016 and 2017, Examine associations with perceptions, behaviors and intentions to consume.	Use of EC as a smoking cessation aid may be declining even more for young people, and EC use initiation followed by smoking initiation appears to be increasing.
Foxon et al.^95^	Longitudinal study	American adolescents aged 12–17 years N=12500 N=31000	Examine the prevalence of exclusive EC use, cigarette use, or dual use and determine the ages of onset.	ECs have a higher age of onset compared to cigarettes. ECs have not increased the overall prevalence of nicotine consumers. ECs have not acted as a risk factor (gateway to cigarette use). The findings are more consistent with the idea that ECs deter adolescents from cigarette use, as ECs are estimated to be less harmful, thus having substantial implications for harm reduction regarding subsequent nicotine use.
Jongelis et al.^79^	Cross-sectional study	N=4617 Australians aged 12–17 years	To investigate susceptibility to EC use among Australian adolescents who had never smoked cigarettes or used ECs.	45% of the sample who had never smoked or vaped were susceptible to future vaping. Important correlates of susceptibility included the belief that EC use helps with smoking cessation.
Kinnunen et al.^72^	Longitudinal study	N=5742 students baseline 2011 (aged 12–13 years), second survey 2014 (aged 15–16 years)	Explore whether EC use with or without nicotine predicts daily use of EC and conventional cigarettes.	Similar risk factors for EC and conventional cigarette and for boys and girls. Low academic performance strongest predictor of EC experimentation (excellent academic performance protects). Parental smoking increased risk of experimenting with EC and smoking, slightly more among girls than boys.
Kinnunen et al.^83^	Longitudinal study	N=3474 students baseline 2014 (aged 15–16 years), second survey 2016 (aged 17–18 years)	To explore if EC use with or without nicotine predicts daily use of EC and conventional cigarettes.	Experimentation with nicotine EC serves as a gateway to later use of conventional cigarettes and nicotine EC.
Kinnunen et al.^52^	Cross-sectional study	N=12167 European students aged 13–18 years (Netherlands, Portugal, Ireland, Germany, Italy, Belgium and Finland)	Identify the social correlates of EC use, and if they are the same for EC-only use, conventional cigarette-only use, and dual use.	ECs appear to be complementary to conventional cigarettes. They are primarily another nicotine product in addition to conventional cigarettes, and not a replacement for them.
Lechner et al.^80^	Longitudinal study	N=1023 American students Average age=12.2 years Survey every 6 months for two years, and one at end of year	To examine the specific role of cognitive susceptibility to smoking prior to initiating use of any of the products, while controlling for other common risk factors.	Cognitive susceptibility to smoking → risk factor for future cigarette or EC use.
Owotomo et al.^86^	Prospective cohort study	N=8661 American adolescents aged 12–17 years	Examine if the likelihood of switching from EC use to cigarettes differs according to the intention of smoking.	EC use was associated with an increased risk of cigarette smoking among those who had no prior intention to smoke → it is plausible that EC users start out with no intention to smoke conventional cigarettes, but develop a nicotine use disorder that creates the intention to smoke and subsequent EC use.
Park et al.^92^	Longitudinal study	N=801 American adolescents aged 13–15 years	To determine if different EC use patterns are associated with alcohol and marijuana use.	Both high and low levels of EC use are associated with increased use of other substances (alcohol and marijuana).
Staff et al.^73^	Longitudinal study	N=11564 English adolescents aged 14 years and their parents	Identify links between EC use, delinquency and other substance use.	Intermediate position of exclusive EC users between non-users and dual/fuel users → emerging ECs pattern of health risk behaviors and maladaptation for some youths.
Vogel et al.^74^	Longitudinal study	N=173 American adolescents aged 13–18 years (consumed at least once in last 30 days and at least 10 times in their lifetime)	Examine the reasons to use and stop using ECs. Determine persistence in EC use and dual consumption and stability in frequency and dependence of EC use.	No dual users abstained from both products at any of the follow-ups → little evidence of a smoking cessation benefit among dual users. Significant increases in frequency of use, nicotine exposure and dependence on ECs. The percentage of daily EC users doubled from 14.5% at baseline to 29.8% at 12-month follow-up.
Vrinten et al.^69^	Longitudinal study	N=9731 English students aged 14 (baseline) to 17 years (follow-up)	Describe patterns of EC and cigarette consumption.	Identified four probabilistic classes: ‘continuous abstainers’, who did not use any product; ‘experimenters’, with low levels of irregular use; ‘late adopters’, with low levels of use at age 14 but higher use at age 17; ‘early adopters’, with high levels of use at both ages 14 and 17. Higher levels of experimentation with ECs than with cigarettes, but low levels of current use. Tobacco and/or EC use by caregivers and smoking among peers were associated with onset of consumption.
Wang et al.^93^	Longitudinal study	N=9692 American adolescents aged 12–16 years who had never used cannabis	To examine the association between EC use and cannabis consumption over a 12-month follow-up period.	EC use was significantly associated with subsequent initiation of cannabis consumption.
Yang et al.^84^	Longitudinal study	N=1977 students initially recruited in 2013 and followed every 6 months	Describe the transition probabilities of EC and cigarette use over time.	ECs were more strongly associated with subsequent initiation of cigarette consumption than vice versa, although the use of either product seems to promote the use of the other. ECs reduced the likelihood of being used as a successful aid to quit smoking for cigarette smokers.
Zavala-Arciniega et al.^56^	Cross-sectional study	N=951 Mexican students aged 12–13 years at baseline who have used in the past month	To assess the correlates of the frequency of EC use among Mexican students who currently use EC.	Few students used CE for smoking cessation (7.5%).

**Figure 6:**
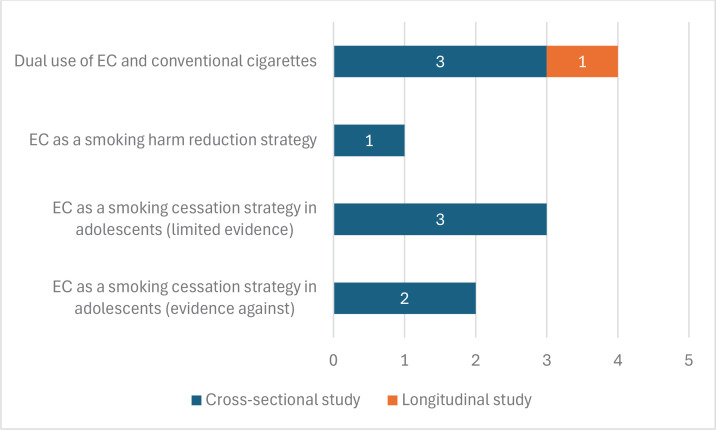
Number of studies analyzed that confirm the relationship between the use of EC and the consumption of conventional cigarettes and other substances

In Figure 6, the number of studies confirming the limited evidence regarding the use of ECs as a smoking cessation strategy in adolescents (with three cross-sectional studies^56,79,82^) or as a harm reduction strategy in smoking (with one cross-sectional study^94^) is presented. Conversely, there are two cross-sectional studies^81,85^ that find evidence against the use of ECs as a smoking cessation strategy in adolescents. In fact, the highest quality evidence, through one longitudinal study^95^ and three cross-sectional studies^47,52^, indicates that EC use is a complementary form of nicotine consumption to conventional cigarettes, with dual use of both modalities observed. All these studies are of low quality according to GRADE^46^.

## DISCUSSION

The present study posed the following research question: ‘What are the risk and protective factors for e-cigarette use among adolescents, its association with tobacco and other substance use, and the evidence of its use as a smoking cessation strategy?’. To address this question, the aim was to determine the current evidence on risk and protective factors, and motives for EC use among adolescents. Additionally, the aim was to investigate whether EC use is associated with increased risk of tobacco smoking and consumption of other substances, and what evidence exists regarding the use of ECs as a harm reduction strategy or for smoking cessation in adolescents.

The findings point out various risk factors repeatedly found in studies and some of the reasons for EC use in adolescents. The evidence on the relationship between EC use and smoking and consumption of other substances is abundant, indicating a positive relationship. And, regarding the use of ECs as a harm reduction strategy or for smoking cessation in adolescents, the evidence is not conclusive. However, most studies are of low quality according to the GRADE criteria, so the evidence should be considered weak, highlighting the need for the development of studies that provide more robust findings.

### Evidence on risk and protective factors and prevention proposals

Concerning risk factors, no changes are evident between the previous and current evidence with respect to gender, with males being the main users^47,48,50-56,59,97^. The existing evidence for sensation seeking is also consistent^59,97^ and so is impulsivity^59,98-100^. However, sensation seeking may not be an important factor in sustained EC use over The analysis of age as a risk or protective factor for the use of ECs is one of the novel findings of most recent studies included in the present study compared to previous literature. However, there is no consensus. Some authors find that older adolescents are less likely to use ECs compared to younger ones^57^, while others find completely the opposite where older adolescents are more at risk^48,52,58,71^. In this regard, all studies are cross-sectional, with variability also in terms of sample size and the country in which they are conducted, which poses a number of limitations for establishing conclusive evidence. Future studies with more robust experimental designs can clarify this issue.

The Framework Convention on Tobacco Control^39^ highlights the concern about tobacco use by children and adolescents, especially highlighting the risk and need for intervention at progressively younger ages. Importantly, those who start smoking in adolescence are at increased risk of becoming daily smokers^100^. Although the long-term effects are still unknown, nicotine exposure during adolescence may have adverse consequences for brain development and could lead to dependence^21,101-103^. Consequently, regardless of the age of adolescents, not using ECs and other forms of tobacco use should be encouraged.

Based on the evidence accumulated and confirmed in the present study, the components that clearly should be incorporated into school-based universal prevention programs are those that address sensation seeking, impulsivity control and the social normalization of consumption. From a theoretical framework, this finding is consistent with the theory of planned behavior^104^. The probability that an adolescent has used an EC is a function of the use of friends (social norms), expectations about costs and benefits (beliefs), and self-efficacy to resist (perceived control). Also related to this, direct social influence (pressure)^105^ as well as indirect (group expectations, modeling, etc.) social influence processes^106^ may play a relevant role in the initiation and maintenance of EC use. Adolescents exposed to ECs report lower self-efficacy to resist use^57^. Consequently, false beliefs related to the normalization of consumption and also on the perception of low risk associated with the use of ECs, a strategy skillfully used by the industry, must be dismantled^60-64,78-80^.

Nevertheless, beyond risk and protective factors, it is important to consider the importance of the vulnerability that these factors create in adolescents and identify individuals or groups that are more vulnerable. Perhaps the future challenge of prevention will be to establish mechanisms for detecting vulnerability to drug use at an early age, and to devise effective responses through selective and appropriate prevention programs aimed not only at these minors but also their immediate environment, such as family and school.

It should not be forgotten that the family plays a relevant role. Parents act as role models, reflecting that smoking is not harmful^50,51,62,65,67,69,72^. In the same way, the positive influence of parents against tobacco consumption can cushion the effect of peer norms and positive expectations in a teenager’s decision to participate in EC use^96^. This suggests that in order to reduce the consumption of tobacco and related products, such as ECs, in the adolescent population, it is not enough to implement preventive programs aimed at them. It is essential to include the social responsibility of the adult community and families. Consequently, public policies should promote measures that have been shown to be effective in reducing smoking in adults from an environment perspective, such as increasing the price of tobacco, restricting consumption in public spaces, restricting consumption in private spaces with the presence of minors, such as cars, sustained information campaigns on tobacco and health, and promoting smoking cessation programs.

In addition, schools play a key role. As noted in previous studies^107^, excellent academic performance is a protective factor against experimentation with ECs^72^. The combination of gender and educational environment has also been found to be a determinant. Being a boy and belonging to schools with high levels of heavy alcohol and marijuana use is an EC use risk factor compared to being a girl in the same school environment^71^. Consequently, schools can contribute in reducing the use of tobacco and related products, as well as other drugs, through school policy that promotes health. This can include measures such as: 1) restricting consumption in internal and surrounding areas, applicable to teachers, students and families; 2) establishing protocols for the detection of consumption and underage consumers, and coordinated management with the family and specific social and health devices; 3) promoting school reinforcement programs for children with poor academic performance; and 4) providing and sustaining drug use prevention programs that have proven their effectiveness, as opposed to the tendency to incorporate pseudo-prevention actions not based on scientific evidence or good practices.

### Evidence on the reasons for EC use and preventive proposals

In the present study, consistent with previous studies^12-14^, the main reasons reported for EC use initiation and consolidation were socialization, enjoyment, flavors^74^ and curiosity^56,60,64,75^, a lesser perception of the negative health consequences of conventional cigarettes^57,61-64,80^. This suggests that ECs, marketed as a healthier and socially acceptable alternative, appeal to an adolescent population that would otherwise engage in positive lifestyle behaviors and not use conventional cigarettes^47^. However, Vogel et al.^74^ indicate a significant increase in the percentage of daily users, frequency of use, nicotine exposure and dependence among adolescents who use ECs.

Therefore, there is a need for adolescents to have better knowledge about EC content, perceptions of harmfulness and addictiveness, as these have all been linked to lower intention to try ECs, and thus prevent or delay the use of ECs or even conventional cigarettes^108^. But it is also necessary to implement the measures contemplated in the Framework Convention on Tobacco Control^39^, especially those pertaining to advertising, advancing legislation that leaves no loopholes for indirect or hidden advertising. Consistent with studies developed in this regard on tobacco^41^ and cannabis^109^, specific actions are suggested for the regulation of direct, indirect and covert advertising through audiovisual media (cinema, series, documentaries), video games, internet, social networks, influencers and dissemination of fake news, and in the sponsorship of leisure events aimed at the adolescent and youth population.

### Evidence on the association of EC use with the consumption of other substances and preventive proposals

The present study reveals the broad consensus that EC use may facilitate smoking initiation^17-24,51,67^ and the need to emphasize once more that ECs may serve as a gateway to subsequent conventional cigarette use^49,81-85^. This is important because it occurs whether ECs are used with nicotine or non-nicotine cartridges. Vaping familiarizes users with the sensorimotor and social components associated with smoking^110^. There are studies that show that adolescents who vape without having used tobacco are more likely to initiate tobacco use than those who have not^21^, and those who vape frequently are more likely to use tobacco 6 months later^111^. In addition, those youths who use conventional tobacco or use ECs are more likely to use cannabis via ECs^112^. In short, it is advisable to denormalize the use of non-nicotine ECs in both adolescent and adult populations, to increase the risk perception of its use. It is not a harmless product, and it behaviorally and contextually predisposes subsequent consumption of ECs with nicotine and conventional tobacco. This objective should be incorporated transversally in prevention with adolescents, families and in those environmental prevention measures aimed at informing and raising awareness among the population about the risks of EC use.

In addition, the evidence that EC users are more likely to use other substances is reinforced by the findings. This is confirmed for alcohol^3,28,48,54,91,92^, cannabis ^28-31,48,88,90-93^ and non-prescription drugs^28,29,30,89,91^. This suggests a substance use progression of more concern for young people using ECs, as nicotine may be priming the adolescent brain for future use of other substances^89^. It may also be due to the presence of risk factors common to the use of different substances. Both perspectives reinforce the idea of adolescents with greater vulnerability, and the need to focus preventive efforts on early detection and early intervention with them and in their immediate environment.

### Evidence on the use of ECs as a smoking cessation strategy and preventive proposals

That ECs are an effective strategy for smoking cessation is the Trojan horse used by the industry to promote the belief among consumers that it is a healthier option than conventional tobacco and the solution for those who wish to quit. However, as cessation strategy, there is clear evidence that it is of little value to adolescents. This is a new finding in most current literature, since the previous studies examined did not refer to the adolescent population^55,56,81,82,84^. In fact, the usefulness of ECs as a nicotine replacement therapy device in the adult population is only confirmed in a very small number of controlled clinical trials and would be conditional on whether the smoker knows how to self-administer the appropriate nicotine dose^36^. This condition is unlikely in the adolescent population, who receive all the pernicious properties of nicotine without the suggested harm reduction benefits^113^. Second, ECs appear to be complementary to conventional cigarettes, meaning that they are a nicotine product in addition to conventional cigarettes, and not a substitute^47,52^. Furthermore, as noted above, frequent use was associated with increased odds of cigarette smoking^49,51,67,82,85^. In fact, ECs and conventional cigarette dual use is the most frequent behavior among adolescent users of nicotine products^47^.

What are the implications of being a dual user of ECs and conventional cigarettes? Evidence indicates that dual users tend to maintain consumption of both products over time^74^. This could be explained by the fact that experiencing more EC-specific dependence symptoms was associated with lower probability of both wanting to quit and attempting to quit in the past year^94^. Also, dual user adolescents may view ECs as less harmful and therefore be less motivated to quit using them^94^.

On the other hand, and in contrast to previous studies^1^, the risk factor profiles of exclusive EC users were found to differ from dual and conventional cigarette users^47,68^. Several studies have found a sequential risk gradient for risk behaviors^73^ specifically for cannabis consumption^88^. But all of these studies^73,88^ agree on the order of user risk led by: 1) dual users with the highest risk; followed by 2) traditional cigarette smokers only; 3) EC users only; and 4) non-users with the lowest risk.

### Limitations

This systematic review is not without limitations. The results obtained cannot establish causal relationships, as essentially more than half of the studies were cross-sectional. At the methodological level, all selected studies were of good methodological quality, although the quality of evidence according to GRADE is low or very low in most cases. This, along with the heterogeneity of study designs, suggests that most findings should be interpreted with caution. Future studies with better experimental designs are needed to confirm the observed findings, providing greater robustness to the evidence regarding the aspects analyzed in relation to EC use. Another limitation of the present study is the lack of a meta-analysis, which could be conducted in the future with higher-quality studies. However, given the geographical diversity of the samples used, the findings are not broadly generalizable as most come from countries belonging to high-income economies.

## CONCLUSIONS

There is a need to improve the quality of the designs of future studies, to obtain more robust evidence. Out of the 50 studies analyzed, only 2 were randomized controlled trials; and among observational studies, 20 were longitudinal studies. Despite these limitations, the direction of the evidence has not substantially changed in recent years, but rather has been reinforced. Regarding the risk factors to adolescents with the strongest empirical support, these include: 1) greater acceptance and use of ECs in their social circles (family or friends); 2) gender, indicating that males have a higher use of ECs; 3) low risk perception; 4) age, where a younger age indicates a higher risk; and 5) greater availability of money among adolescents. The consumption motives most supported by the evidence found in this study are flavors and the perception of low risk. Regarding the use of ECs and cigarette consumption and initiation of smoking, the evidence found confirms previous literature. It also confirms its relationship with the consumption of cannabis and alcohol. The evidence on the use of ECs as a strategy to reduce harm or quit smoking in adolescents is contradictory. In fact, the evidence indicates that the use of ECs is a complementary form of nicotine consumption to conventional cigarettes, with dual use of both being observed.

Based on these findings, it is clear that tobacco consumption and the use of ECs are not an individual responsibility attributable to adolescents, but rather the consequence of an accumulation of factors that make them vulnerable to consumption. This implies a community approach, with preventive policies at the state and municipal levels involving all social and educational agents, with special emphasis on the family, especially those who are also more vulnerable to tobacco consumption.

## Supplementary Material



## Data Availability

Data sharing is not applicable to this article as no new data were created.

## References

[1] Hanewinkel R, Isensee B. Risk factors for e-cigarette, conventional cigarette, and dual use in German adolescents: a cohort study. Prev Med. 2015;74:59-62. doi:10.1016/j.ypmed.2015.03.006 25770433

[2] Glasser AM, Collins L, Pearson JL, et al. Overview of electronic nicotine delivery systems: a systematic review. Am J Prev Med. 2017;52(2):e33-e66. doi:10.1016/j.amepre.2016.10.036 27914771 PMC5253272

[3] Dunbar MS, Tucker JS, Ewing BA, et al. Frequency of e-cigarette use, health status, and risk and protective health behaviors in adolescents. J Addict Med. 2017;11(1):55-62. doi:10.1097/ADM.0000000000000272 27898495 PMC5291796

[4] Fite PJ, Cushing CC, Poquiz J, Frazer AL. Family influences on the use of e-cigarettes. Journal of Substance Use. 2018;23(4);396-401. doi:10.1080/14659891.2018.1436601

[5] Fadus MC, Smith TT, Squeglia LM. The rise of e-cigarettes, pod mod devices, and JUUL among youth: factors influencing use, health implications, and downstream effects. Drug Alcohol Depend. 2019;201:85-93. doi:10.1016/j.drugalcdep.2019.04.011 31200279 PMC7183384

[6] Krishnan-Sarin S, Morean ME, Camenga DR, Cavallo DA, Kong G. E-cigarette use among high school and middle school adolescents in Connecticut. Nicotine Tob Res. 2015;17(7):810-818. doi:10.1093/ntr/ntu243 25385873 PMC4674435

[7] Budney AJ, Sargent JD, Lee DC. Vaping cannabis (marijuana): parallel concerns to e-cigs? Addiction. 2015;110(11):1699-1704. doi:10.1111/add.13036 26264448 PMC4860523

[8] Coleman BN, Apelberg BJ, Ambrose BK, et al. Association between electronic cigarette use and openness to cigarette smoking among US young adults. Nicotine Tob Res. 2015;17(2):212-218. doi:10.1093/ntr/ntu211 25378683 PMC4892708

[9] Lucherini M, Rooke C, Amos A. E-cigarettes, vaping and performativity in the context of tobacco denormalisation. Sociol Health Illn. 2018;40(6):1037-1052. doi:10.1111/1467-9566.12741 29664119 PMC6055866

[10] Tan CE, Kyriss T, Glantz SA. Tobacco company efforts to influence the food and drug administration-commissioned Institute of Medicine report clearing the smoke: an analysis of documents released through litigation. PLoS Med. 2013;10(5):e1001450. doi:10.1371/journal.pmed.1001450 23723740 PMC3665841

[11] Ali M, Gray TR, Martinez DJ, Curry LE, Horn KA. Risk profiles of youth single, dual, and poly tobacco users. Nicotine Tob Res. 2016;18(7):1614-1621. doi:10.1093/ntr/ntw028 26896162

[12] Bold KW, Kong G, Cavallo DA, Camenga DR, Krishnan-Sarin S. Reasons for trying e-cigarettes and risk of continued use. Pediatrics. 2016;138(3):e20160895. doi:10.1542/peds.2016-0895 27503349 PMC5005025

[13] Patrick ME, Miech RA, Carlier C, O’Malley PM, Johnston LD, Schulenberg JE. Self-reported reasons for vaping among 8th, 10th, and 12th graders in the US: Nationally-representative results. Drug Alcohol Depend. 2016;165:275-278. doi:10.1016/j.drugalcdep.2016.05.017 27286951 PMC4939118

[14] Tsai J, Walton K, Coleman BN, et al. Reasons for electronic cigarette use among middle and high school students - National Youth Tobacco Survey, United States, 2016. MMWR Morb Mortal Wkly Rep. 2018;67(6):196-200. doi:10.15585/mmwr.mm6706a5 29447148 PMC5815490

[15] Choi K, Forster JL. Beliefs and experimentation with electronic cigarettes: a prospective analysis among young adults. Am J Prev Med. 2014;46(2):175-178. doi:10.1016/j.amepre.2013.10.007 24439352 PMC3930913

[16] Saddleson ML, Kozlowski LT, Giovino GA, et al. Risky behaviors, e-cigarette use and susceptibility of use among college students. Drug Alcohol Depend. 2015;149:25-30. doi:10.1016/j.drugalcdep.2015.01.001 25666362

[17] Berry KM, Fetterman JL, Benjamin EJ, et al. Association of electronic cigarette use with subsequent initiation of tobacco cigarettes in US youths. JAMA Netw Open. 2019;2(2):e187794. doi:10.1001/jamanetworkopen.2018.7794 30707232 PMC6484602

[18] Bold KW, Sussman S, O’Malley SS, et al. Measuring e-cigarette dependence: initial guidance. Addict Behav. 2018;79:213-218. doi:10.1016/j.addbeh.2017.11.015 29174664 PMC5807200

[19] Hammond D, Reid JL, Cole AG, Leatherdale ST. Electronic cigarette use and smoking initiation among youth: a longitudinal cohort study. CMAJ. 2017;189(43):E1328-E1336. doi:10.1503/cmaj.161002 29084759 PMC5662449

[20] Lozano P, Barrientos-Gutierrez I, Arillo-Santillan E, et al. A longitudinal study of electronic cigarette use and onset of conventional cigarette smoking and marijuana use among Mexican adolescents. Drug Alcohol Depend. 2017;180:427-430. doi:10.1016/j.drugalcdep.2017.09.001 28988005 PMC5771440

[21] Primack BA, Soneji S, Stoolmiller M, Fine MJ, Sargent JD. Progression to traditional cigarette smoking after electronic cigarette use among US adolescents and young adults. JAMA Pediatr. 2015;169(11):1018-1023. doi:10.1001/jamapediatrics.2015.1742 26348249 PMC4800740

[22] Soneji S, Barrington-Trimis JL, Wills TA, et al. Association between initial use of e-cigarettes and subsequent cigarette smoking among adolescents and young adults: a systematic review and meta-analysis. JAMA Pediatr. 2017;171(8):788-797. doi:10.1001/jamapediatrics.2017.1488 28654986 PMC5656237

[23] Watkins SL, Glantz SA, Chaffee BW. Association of noncigarette tobacco product use with future cigarette smoking among youth in the population assessment of tobacco and health (PATH) study, 2013-2015. JAMA Pediatr. 2018;172(2):181-187. doi:10.1001/jamapediatrics.2017.4173 29297010 PMC5801043

[24] Wills TA, Knight R, Sargent JD, Gibbons FX, Pagano I, Williams RJ. Longitudinal study of e-cigarette use and onset of cigarette smoking among high school students in Hawaii. Tob Control. 2017;26(1):34-39. doi:10.1136/tobaccocontrol-2015-052705 26811353 PMC4959970

[25] Aleyan S, Cole A, Qian W, Leatherdale ST. Risky business: a longitudinal study examining cigarette smoking initiation among susceptible and non-susceptible e-cigarette users in Canada. BMJ Open. 2018;8(5):e021080. doi:10.1136/bmjopen-2017-021080 PMC5988055 29804064

[26] Pénzes M, Foley KL, Nădășan V, Paulik E, Ábrám Z, Urbán R. Bidirectional associations of e-cigarette, conventional cigarette and waterpipe experimentation among adolescents: a cross-lagged model. Addict Behav. 2018;80:59-64. doi:10.1016/j.addbeh.2018.01.010 29355818 PMC5807159

[27] Treur JL, Rozema AD, Mathijssen JJP, van Oers H, Vink JM. E-cigarette and waterpipe use in two adolescent cohorts: cross-sectional and longitudinal associations with conventional cigarette smoking. Eur J Epidemiol. 2018;33(3):323-334. doi:10.1007/s10654-017-0345-9 29260431 PMC5889768

[28] Milicic S, Leatherdale ST. The associations between E-cigarettes and binge drinking, marijuana use, and energy drinks mixed with alcohol. J Adolesc Health. 2017;60(3):320-327. doi:10.1016/j.jadohealth.2016.10.011 28012834

[29] Dai H, Hao J. Electronic cigarette and marijuana use among youth in the United States. Addict Behav. 2017;66:48-54. doi:10.1016/j.addbeh.2016.11.005 27871045

[30] Mehra VM, Keethakumar A, Bohr YM, Abdullah P, Tamim H. The association between alcohol, marijuana, illegal drug use and current use of E-cigarette among youth and young adults in Canada: results from Canadian Tobacco, Alcohol and Drugs Survey 2017. BMC Public Health. 2019;19(1):1208. doi:10.1186/s12889-019-7546-y 31477067 PMC6721192

[31] Nicksic NE, Barnes AJ. Is susceptibility to e-cigarettes among youth associated with tobacco and other substance use behaviors one year later? Results from the PATH study. Prev Med. 2019;121:109-114. doi:10.1016/j.ypmed.2019.02.006 30776386 PMC6594855

[32] Goniewicz ML, Knysak J, Gawron M, et al. Levels of selected carcinogens and toxicants in vapour from electronic cigarettes. Tob Control. 2014;23(2):133-139. doi:10.1136/tobaccocontrol-2012-050859 23467656 PMC4154473

[33] Polosa R, Morjaria JB, Prosperini U, et al. Health effects in COPD smokers who switch to electronic cigarettes: a retrospective-prospective 3-year follow-up. Int J Chron Obstruct Pulmon Dis. 2018;13:2533-2542. doi:10.2147/COPD.S161138 30197510 PMC6113943

[34] Bullen C, Howe C, Laugesen M, et al. Electronic cigarettes for smoking cessation: a randomised controlled trial. Lancet. 2013;382(9905):1629-1637. doi:10.1016/S0140-6736(13)61842-5 24029165

[35] Buu A, Hu YH, Piper ME, Lin HC. The association between e-cigarette use characteristics and combustible cigarette consumption and dependence symptoms: results from a national longitudinal study. Addict Behav. 2018;84:69-74. doi:10.1016/j.addbeh.2018.03.035 29627636 PMC5975121

[36] McRobbie H, Bullen C, Hartmann-Boyce J, Hajek P. Electronic cigarettes for smoking cessation and reduction. Cochrane Database Syst Rev. 2014;(12):CD010216. doi:10.1002/14651858.CD010216.pub2 25515689

[37] Fulton E, Gokal K, Griffiths S, Wild S. More than half of adolescent e-cigarette users had never smoked a cigarette: findings from a study of school children in the UK. Public Health. 2018;161:33-35. doi:10.1016/j.puhe.2018.04.014 29870832

[38] Observatorio Español de las Drogas y las Adicciones. Report 2021. Alcohol, tobacco and illegal drugs in Spain. Informe 2021: Alcohol, tabaco y drogas ilegales en España. Ministerio de Sanidad, Delegación del Gobierno para el Plan Nacional sobre Drogas; 2021. Accessed December 1, 2024. https://pnsd.sanidad.gob.es/profesionales/sistemasInformacion/informesEstadisticas/pdf/2021OEDA-INFORME.pdf

[39] World Health Organization. Resolutions: WHA56.1. Accessed December 1, 2024. https://apps.who.int/gb/ebwha/pdf_files/WHA56/ea56r1.pdf

[40] World Health Organization. MPOWER: A plan of measures to roll back the tobacco epidemic. MPOWER: un plan de medidas para hacer retroceder la epidemia de tabaquismo. World Health Organization; 2008. Accessed December 1, 2024. https://iris.who.int/bitstream/handle/10665/43891/9789243596631_spa.pdf?sequence=1&isAllowed=y

[41] Isorna Folgar M, de la Cruz Amorós E, Villanueva-Blasco VJ. Tobacco violence: the role of audiovisual media, influencers and think tanks. La violencia tabáquica: papel de los medios audiovisuales, influencers y las think tanks. Revista Española de Drogodependencias. 2020;45(1):101-110. Accessed December 1, 2024. https://www.aesed.com/upload/files/v45n1-8_misorna.pdf

[42] Feliu A, Martínez C, Fernández E. Lights and shadows for public health: a critical analysis of the tobacco legislation in Spain. Luces y sombras para la salud pública: análisis crítico de la legislación sobre el tabaco en España. Gac Sanit. 2022;36(1):48-52. doi:10.1016/j.gaceta.2021.07.001 34419288

[43] Villalbí JR, Suelves JM, Martínez C, Valverde A, Cabezas C, Fernández E. Smoking control in Spain: current situation and priorities. El control del tabaquismo en España: situación actual y prioridades. Rev Esp Salud Pública. 2019;93:1-16. Accessed December 1, 2024. https://scielo.isciii.es/pdf/resp/v93/1135-5727-resp-93-e201907044.pdf PMC10308847 31298227

[44] Page MJ, McKenzie JE, Bossuyt PM, et al. Updating guidance for reporting systematic reviews: development of the PRISMA 2020 statement. J Clin Epidemiol. 2021;134:103-112. doi:10.1016/j.jclinepi.2021.02.003 33577987

[45] Hong QN, Fàbregues S, Bartlett G, et al. The mixed methods appraisal tool (MMAT) version 2018 for information professionals and researchers. Education for Information. 2018; 34(4):285-291. doi:10.3233/EFI-180221

[46] Aguayo-Albasini JL, Flores-Pastor B, Soria-Aledo V. GRADE system: classification of quality of evidence and strength of recommendation. Sistema GRADE: clasificación de la calidad de la evidencia y graduación de la fuerza de la recomendación. Cir Esp. 2014;92(2):82-88. doi:10.1016/j.ciresp.2013.08.002 24361098

[47] Bowe AK, Doyle F, Stanistreet D, et al. E-cigarette-only and dual use among adolescents in Ireland: emerging behaviours with different risk profiles. Int J Environ Res Public Health. 2021;18(1):332. doi:10.3390/ijerph18010332 33466304 PMC7795664

[48] Chang YP, Seo YS. E-cigarette use and concurrent risk behaviors among adolescents. Nurs Outlook. 2021;69(3):302-310. doi:10.1016/j.outlook.2020.09.005 33121761

[49] Conner M, Grogan S, Simms-Ellis R, et al. Association between age at first reported e-cigarette use and subsequent regular e-cigarette, ever cigarette and regular cigarette use. Addiction. 2021;116(7):1839-1847. doi:10.1111/add.15386 33394523 PMC8609424

[50] Dai L, Lu W, Wang J, Zhang L, Zhu J. Social environment exposure to electronic cigarettes and its association with e-cigarette use among adolescents in Shanghai, China. Front Public Health. 2022;10:1005323. doi:10.3389/fpubh.2022.1005323 36407975 PMC9669338

[51] Fan J, Mao T, Zhen S, Xu Y, Qu C. Comparative analysis of e-cigarette prevalence and influencing factors among adolescents in Jiangsu Province, China. Front Public Health. 2023;11:1221334. doi:10.3389/fpubh.2023.1221334 38106882 PMC10722425

[52] Kinnunen JM, Rimpelä AH, Lindfors PL, et al. Electronic cigarette use among 14- to 17-year-olds in Europe. Eur J Public Health. 2021;31(2):402-408. doi:10.1093/eurpub/ckaa145 33079986 PMC8071596

[53] Tarasenko Y, Ciobanu A, Fayokun R, Lebedeva E, Commar A, Mauer-Stender K. Electronic cigarette use among adolescents in 17 European study sites: findings from the Global Youth Tobacco Survey. Eur J Public Health. 2022;32(1):126-132. doi:10.1093/eurpub/ckab180 34694383 PMC8807119

[54] Van Minh H, Long KQ, Van Vuong D, et al. Tobacco and electronic cigarette smoking among in-school adolescents in Vietnam between 2013 and 2019: prevalence and associated factors. Glob Health Action. 2022;15(1):2114616. doi:10.1080/16549716.2022.2114616 36174100 PMC9542268

[55] Wamba A, Nekaa M, Leclerc L, Denis-Vatant C, Masson J, Pourchez J. Regional French evolution of tobacco and e-cigarette experimentation and use among adolescents aged 15-16 years: a cross-sectional observational study conducted in the Loire department from 2018 to 2020. Prev Med Rep. 2023;35:102278. doi:10.1016/j.pmedr.2023.102278 37389205 PMC10300395

[56] Zavala-Arciniega L, Lozano P, Kollath-Cattano C, et al. E-cigarette use frequency and motivations among current users in middle school. Drug Alcohol Depend. 2019;204:107585. doi:10.1016/j.drugalcdep.2019.107585 31590130 PMC6944441

[57] Durkin K, Williford DN, Turiano NA, et al. Associations between peer use, costs and benefits, self-efficacy, and adolescent e-cigarette use. J Pediatr Psychol. 2021;46(1):112-122. doi:10.1093/jpepsy/jsaa097 33120416 PMC8456300

[58] Usidame B, Hirschtick JL, Mattingly DT, Patel A, Patrick ME, Fleischer NL. Sociodemographic patterns of exclusive and dual combustible tobacco and e-cigarette use among US adolescents-A nationally representative study (2017-2020). Int J Environ Res Public Health. 2022;19(5):2965. doi:10.3390/ijerph19052965 35270656 PMC8910207

[59] Conner M, Grogan S, Simms-Ellis R, et al. Patterns and predictors of e-cigarette, cigarette and dual use uptake in UK adolescents: evidence from a 24-month prospective study. Addiction. 2019;114(11):2048-2055. doi:10.1111/add.14723 31254419 PMC6852175

[60] Zhao S, Li Z, Zhang L, et al. The characteristics and risk factors of e-cigarette use among adolescents in Shanghai: a case-control study. Tob Induc Dis. 2023;21(June):83. doi:10.18332/tid/166131 37342865 PMC10277906

[61] Carey FR, Rogers SM, Cohn EA, Harrell MB, Wilkinson AV, Perry CL. Understanding susceptibility to e-cigarettes: a comprehensive model of risk factors that influence the transition from non-susceptible to susceptible among e-cigarette naïve adolescents. Addict Behav. 2019;91:68-74. doi:10.1016/j.addbeh.2018.09.002 30241775 PMC6398945

[62] El-Amin S, Kinnunen JM, Rimpelä A. Adolescents’ perceptions of harmfulness of tobacco and tobacco-like products in Finland. Int J Environ Res Public Health. 2022;19(3):1485. doi:10.3390/ijerph19031485 35162508 PMC8834861

[63] Monzón J, Islam F, Mus S, Thrasher JF, Barnoya J. Effects of tobacco product type and characteristics on appeal and perceived harm: results from a discrete choice experiment among Guatemalan adolescents. Prev Med. 2021;148:106590. doi:10.1016/j.ypmed.2021.106590 33930431 PMC8645268

[64] Tudor TE, Lotrean LM. Opinions and practices regarding electronic cigarette use among middle school students from rural areas of romania. Int J Environ Res Public Health. 2022;19(12):7372. doi:10.3390/ijerph19127372 35742620 PMC9223877

[65] Ahuja N, Kedia SK, Jiang Y, et al. Factors associated with e-cigarette quitting behavior among adolescents in the United States: a prospective observational study. Adolesc Health. 2022;71(6):729-736. doi:10.1016/j.jadohealth.2022.07.001 36088234

[66] Hunter E, Gardner LA, O’Dean S, et al. Peer-related correlates of e-cigarette use in Australian adolescents: a cross-sectional examination. Int J Ment Health Addiction. 2023. doi:10.1007/s11469-023-01200-0

[67] Liu PI, Lin MN, Ho PS, et al. Prediction and potential risk factors for electronic cigarette use behaviors among adolescents: a pilot study in Chiayi, Taiwan. Front Public Health. 2023;11:1140615. doi:10.3389/fpubh.2023.1140615 37397731 PMC10311257

[68] Vogel EA, Ramo DE, Rubinstein ML. Prevalence and correlates of adolescents’ e-cigarette use frequency and dependence. Drug Alcohol Depend. 2018;188:109-112. doi:10.1016/j.drugalcdep.2018.03.051 29763848 PMC5999577

[69] Vrinten C, Parnham JC, Radó MK, et al. Patterns of cigarette and e-cigarette use among UK adolescents: a latent class analysis of the Millennium Cohort Study. Eur J Public Health. 2023;33(5):857-863. doi:10.1093/eurpub/ckad124 37573139 PMC10567249

[70] Ollila H, Tarasenko Y, Ciobanu A, Lebedeva E, Raitasalo K. Exclusive and dual use of electronic cigarettes among European youth in 32 countries with different regulatory landscapes. Tob Control. 2024;33:622-627. doi:10.1136/tc-2022-057749 37185883 PMC11503162

[71] McCabe SE, Boyd CJ, Evans-Polce RJ, McCabe VV, Veliz PT. School-level prevalence and predictors of e-cigarette use in 8th, 10th, and 12th grade U.S. youth: results from a national survey (2015–2016). J Adolesc Health. 2020;67(4):531-541. doi:10.1016/j.jadohealth.2020.03.032 32402800 PMC7723318

[72] Kinnunen JM, Ollila H, Minkkinen J, Lindfors PL, Rimpelä AH. A longitudinal study of predictors for adolescent electronic cigarette experimentation and comparison with conventional smoking. Int J Environ Res Public Health. 2018;15(2):305. doi:10.3390/ijerph15020305 29425188 PMC5858374

[73] Staff J, Maggs JL, Seto C, Dillavou J, Vuolo M. Electronic and combustible cigarette use in adolescence: links with adjustment, delinquency, and other substance use. Adolesc Health. 2020;66(1):39-47. doi:10.1016/j.jadohealth.2019.08.030 PMC6928420 31711837

[74] Vogel EA, Prochaska JJ, Ramo DE, Andres J, Rubinstein ML. Adolescents’ e-cigarette use: increases in frequency, dependence, and nicotine exposure over 12 Months. J Adolesc Health. 2019;64(6):770-775. doi:10.1016/j.jadohealth.2019.02.019 31122507 PMC6538303

[75] Thoonen KAHJ, Jongenelis MI. Motivators of e-cigarette use among Australian adolescents, young adults, and adults. Soc Sci Med. 2024;340:116411. doi:10.1016/j.socscimed.2023.116411 37989045

[76] Leventhal AM, Goldenson NI, Cho J, et al. Flavored e-cigarette use and progression of vaping in adolescents. Pediatrics. 2019;144(5):e20190789. doi:10.1542/peds.2019-0789 31659004 PMC6856781

[77] Davis DR, Morean ME, Bold KW, et al. Cooling e-cigarette flavors and the association with e-cigarette use among a sample of high school students. PLoS One. 2021;16(9):e0256844. doi:10.1371/journal.pone.0256844 34469460 PMC8409641

[78] Thoonen KAHJ, Jongenelis MI. Perceptions of e-cigarettes among Australian adolescents, young adults, and adults. Addict Behav. 2023;144:107741. doi:10.1016/j.addbeh.2023.107741 37121085

[79] Jongenelis MI, Thoonen KAHJ. Factors associated with susceptibility to e-cigarette use among Australian adolescents. Int J Drug Policy. 2023;122:104249. doi:10.1016/j.drugpo.2023.104249 37918316

[80] Lechner WV, Murphy CM, Colby SM, Janssen T, Rogers ML, Jackson KM. Cognitive risk factors of electronic and combustible cigarette use in adolescents. Addict Behav. 2018;82:182-188. doi:10.1016/j.addbeh.2018.03.006 29549801 PMC5881577

[81] Chaffee BW, Watkins SL, Glantz SA. Electronic cigarette use and progression from experimentation to established smoking. Pediatrics. 2018;141(4):e20173594. doi:10.1542/peds.2017-3594 29507167 PMC5869336

[82] Evans-Polce RJ, Veliz P, Boyd CJ, McCabe SE. Initiation patterns and trends of E-cigarette and cigarette use among U.S. adolescents. J Adolesc Health. 2020;66(1):27-33. doi:10.1016/j.jadohealth.2019.07.002 31521510 PMC6928393

[83] Kinnunen JM, Ollila H, Minkkinen J, Lindfors PL, Timberlake DS, Rimpelä AH. Nicotine matters in predicting subsequent smoking after e-cigarette experimentation: A longitudinal study among Finnish adolescents. Drug Alcohol Depend. 2019;201:182-187. doi:10.1016/j.drugalcdep.2019.04.019 31238240

[84] Yang Z, Berhane K, Leventhal AM, Liu M, Barrington-Trimis JL, Thomas DC. Modeling the longitudinal transitions of electronic cigarettes and conventional cigarettes with time-dependent covariates among adolescents. Prev Med. 2022;164:107294. doi:10.1016/j.ypmed.2022.107294 36216121 PMC10002430

[85] Azagba S, Kah K, Latham K. Frequency of e-cigarette use and cigarette smoking among Canadian students. Prev Med. 2019;126:105769. doi:10.1016/j.ypmed.2019.105769 31310786

[86] Owotomo O, Stritzel H, McCabe SE, Boyd CJ, Maslowsky J. Smoking intention and progression from e-cigarette use to cigarette smoking. Pediatrics. 2020;146(6):e2020002881. doi:10.1542/peds.2020-002881 33168672 PMC7781200

[87] Audrain-McGovern J, Stone MD, Barrington-Trimis J, Unger JB, Leventhal AM. Adolescent e-cigarette, hookah, and conventional cigarette use and subsequent marijuana use. Pediatrics. 2018;142(3):e20173616. doi:10.1542/peds.2017-3616 30082450 PMC6317758

[88] Azagba S. E-cigarette use, dual use of e-cigarettes and tobacco cigarettes, and frequency of cannabis use among high school students. Addict Behav. 2018;79:166-170. doi:10.1016/j.addbeh.2017.12.028 29291507

[89] Bentivegna K, Atuegwu NC, Oncken C, DiFranza JR, Mortensen EM. Electronic cigarettes associated with incident and polysubstance use among youth. J Adolesc Health. 2021;68(1):123-129. doi:10.1016/j.jadohealth.2020.05.026 32641242

[90] Duan Z, Wang Y, Weaver SR, et al. Effect modification of legalizing recreational cannabis use on the association between e-cigarette use and future cannabis use among US adolescents. Drug Alcohol Depend. 2022;233:109260. doi:10.1016/j.drugalcdep.2021.109260 35152099 PMC8957562

[91] Evans-Polce RJ, Patrick ME, McCabe SE, Miech RA. Prospective associations of e-cigarette use with cigarette, alcohol, marijuana, and nonmedical prescription drug use among US adolescents. Drug Alcohol Depend. 2020;216:108303. doi:10.1016/j.drugalcdep.2020.108303 32987363 PMC7606638

[92] Park E, Livingston JA, Wang W, Kwon M, Eiden RD, Chang YP. Adolescent e-cigarette use trajectories and subsequent alcohol and marijuana use. Addict Behav. 2020;103:106213. doi:10.1016/j.addbeh.2019.106213 31862618 PMC6954975

[93] Wang Y, Duan Z, Self-Brown SR, et al. Longitudinal associations between e-cigarette use and onset of multiple modes of cannabis use among US adolescents. Addict Behav. 2022;131:107316. doi:10.1016/j.addbeh.2022.107316 35364398 PMC9086173

[94] Case KR, Mantey DS, Creamer MR, Harrell MB, Kelder SH, Perry CL. E-cigarette- specific symptoms of nicotine dependence among Texas adolescents. Addict Behav. 2018;84:57-61. doi:10.1016/j.addbeh.2018.03.032 29627634 PMC6055516

[95] Foxon F, Selya AS. Electronic cigarettes, nicotine use trends and use initiation ages among US adolescents from 1999 to 2018. Addiction. 2020;115(12):2369-2378. doi:10.1111/add.15099 32335976 PMC7606254

[96] Trucco EM, Cristello JV, Sutherland MT. Do parents still matter? the impact of parents and peers on adolescent electronic cigarette use. J Adolesc Health. 2021;68(4):780-786. doi:10.1016/j.jadohealth.2020.12.002 33431246 PMC8012253

[97] Yoong SL, Hall A, Leonard A, et al. Prevalence of electronic nicotine delivery systems and electronic non-nicotine delivery systems in children and adolescents: a systematic review and meta-analysis. Lancet Public Health. 2021;6(9):e661-e673. doi:10.1016/S2468-2667(21)00106-7 34274048 PMC8390387

[98] Case KR, Harrell MB, Pérez A, et al. The relationships between sensation seeking and a spectrum of e-cigarette use behaviors: cross-sectional and longitudinal analyses specific to Texas adolescents. Addict Behav. 2017;73:151-157. doi:10.1016/j.addbeh.2017.05.007 28521240 PMC5523408

[99] Becker TD, Arnold MK, Ro V, Martin L, Rice TR. Systematic review of electronic cigarette use (vaping) and mental health comorbidity among adolescents and young adults. Nicotine Tob Res. 2021;23(3):415-425. doi:10.1093/ntr/ntaa171 32905589

[100] Bold KW, Morean ME, Kong G, et al. Early age of e-cigarette use onset mediates the association between impulsivity and e-cigarette use frequency in youth. Drug Alcohol Depend. 2017;181:146-151. doi:10.1016/j.drugalcdep.2017.09.025 29055268 PMC5683935

[101] Moss HB, Chen CM, Yi HY. Early adolescent patterns of alcohol, cigarettes, and marijuana polysubstance use and young adult substance use outcomes in a nationally representative sample. Drug Alcohol Depend. 2014;136:51-62. doi:10.1016/j.drugalcdep.2013.12.011 24434016

[102] Collaco JM, Drummond MB, McGrath-Morrow SA. Electronic cigarette use and exposure in the pediatric population. JAMA Pediatr. 2015;169(2):177-182. doi:10.1001/jamapediatrics.2014.2898 25546699 PMC5557497

[103] Lanza ST, Vasilenko SA. New methods shed light on age of onset as a risk factor for nicotine dependence. Addict Behav. 2015;50:161-164. doi:10.1016/j.addbeh.2015.06.024 26151579 PMC4519837

[104] Ajzen I. From Intentions to Actions: A Theory of Planned Behavior. In: Kuhl J, Beckmann J, eds. Action Control. SSSP Springer Series in Social Psychology. Springer; 1985. doi:10.1007/978-3-642-69746-3_2

[105] McDonough MH, Jose PE, Stuart J. Bi-directional effects of peer relationships and adolescent substance use: a longitudinal study. J Youth Adolesc. 2016;45(8):1652-1663. doi:10.1007/s10964-015-0355-4 26391360

[106] Gazis N, Connor JP, Ho R. Cultural identity and peer influence as predictors of substance use among culturally diverse Australian adolescents. The Journal of Early Adolescence. 2010;30(3):345-368. doi:10.1177/0272431609333276

[107] Demissie Z, Everett Jones S, Clayton HB, King BA. Adolescent risk behaviors and use of electronic vapor products and cigarettes. Pediatrics. 2017;139(2):e20162921. doi:10.1542/peds.2016-2921 28115539 PMC10962496

[108] Gaiha SM, Duemler A, Silverwood L, Razo A, Halpern-Felsher B, Walley SC. School-based e-cigarette education in Alabama: impact on knowledge of e-cigarettes, perceptions and intent to try. Addict Behav. 2021;112:106519. doi:10.1016/j.addbeh.2020.106519 32890911

[109] Isorna Folgar M, Burillo-Putze G, Villanueva-Blasco VJ. Corporate capture, fake news procannabis and position of consumers before its regulation. Captura corporativa, fake news procannabis y posición de los consumidores ante su regulación. Global Health Promotion. 2023; 95-104.

[110] González-Roz A, Secades-Villa R, Weidberg S. Evaluating nicotine dependence levels in e-cigarette users. Evaluación de los niveles de dependencia de la nicotina en usuarios de cigarrillos electrónicos. Adicciones. 2017;29(2):136-138. doi:10.20882/adicciones.905 28170058

[111] Leventhal AM, Strong DR, Kirkpatrick MG, et al. Association of electronic cigarette use with initiation of combustible tobacco product smoking in early adolescence. JAMA. 2015;314(7):700-707. doi:10.1001/jama.2015.8950 26284721 PMC4771179

[112] Kowitt SD, Osman A, Meernik C, et al. Vaping cannabis among adolescents: prevalence and associations with tobacco use from a cross-sectional study in the USA. BMJ Open. 2019;9(6):e028535. doi:10.1136/bmjopen-2018-028535 PMC6585821 31196904

[113] Ferkol TW, Farber HJ, La Grutta S, et al; Forum of International Respiratory Societies. Electronic cigarette use in youths: a position statement of the Forum of International Respiratory Societies. Eur Respir J. 2018;51(5):1800278. doi:10.1183/13993003.00278-2018 29848575

